# Evaluation of public health surveillance system performance in Dangila district, Northwest Ethiopia: a concurrent embedded mixed quantitative/qualitative facility-based cross-sectional study

**DOI:** 10.1186/s12889-019-7724-y

**Published:** 2019-10-22

**Authors:** Tefera Alemu, Hordofa Gutema, Seid Legesse, Tadesse Nigussie, Yirga Yenew, Kindie Gashe

**Affiliations:** 1 Amhara Public Health Institute, Public Health Emergency Management Directorate, Dessie, Ethiopia; 2 Department of Health Promotion and Behavioural Sciences, School of Public Health, College of Medicine and Health Sciences, Bahir Dar University, Bahir Dar, Ethiopia; 3 Amhara Public Health Institute, Research and Technology Transfer Directorate, Dessie, Ethiopia; 4 Departement of public health, College of Health Science, Mizan Tepi University, Mizan Teferi, Ethiopia; 5 Kobo District Health Office, Kobo, Ethiopia; 6 The Carter Center Ethiopia Injibara Project Office, Injibara, Ethiopia

**Keywords:** Evaluation, Surveillance system, Performance, Dangila, Ethiopia

## Abstract

**Background:**

Evaluation of a surveillance system should be conducted on regular bases to ensure that the system is working as envisioned or not. Therefore, we evaluated Dangila district’s public health surveillance system performance in line with its objectives.

**Methods:**

In August 2017, a concurrent embedded mixed quantitative/qualitative, facility-based cross-sectional study was conducted in Dangila district among 12 health facilities/sites. The qualitative part involved 12 purposively selected key stakeholders interview. A semi-structured questionnaire adapted from updated CDC guideline for evaluating public health surveillance system was used for data collection through face to face interview and record review. The major qualitative findings were narrated and summarized based on thematic areas to supplement the quantitative findings. The quantitative findings were analyzed using Microsoft Excel 2007.

**Results:**

All necessary surveillance guidelines, registers and reporting formats were distributed adequately to health facilities. Only the district health office has Emergency Preparedness and Response Plan (EPRP), but not supported by the budget required to respond in case an emergency occurred. There were no regular data analysis and interpretations in terms of time, place and person. Weekly report completeness and timeliness were 100 and 94.6% respectively. The information collected was considered relevant by its users to detect outbreaks early with high acceptability. All stakeholders agreed that the system is simple, easy to understand, representative and can accommodate modifications. Written feedbacks were not obtained in all health facilities. The supervision checklist obtained in the district was not adequate to assess surveillance activities in detail. The calculated positive predictive value for malaria was 11%.

**Conclusions:**

The surveillance system was simple, useful, flexible, acceptable and representative. Report completeness and timelines were above the national and international targets. However, the overall implementation of the system in the district was not satisfactory to achieve the intended objective of surveillance for public health action due to the lack of regular data analysis and feedback dissemination. To create a well-performing surveillance system, regular supervision and epidemiologically analyzed and interpreted feedback system is mandatory.

## Introduction

Public health surveillance is the continuous and systematic data collection, analysis, interpretation and dissemination regarding diseases or other health-related events that present a potential threat to public health security. It primarily aims to prevent and control diseases/conditions under surveillance and thereby to improve health [1, 2]. The system is designed to monitor routine and ad hoc data within and outside the health system and to use them to assess risks to public health. If predefined alert or action thresholds are surpassed, the system triggers rapid response activities [3]. Moreover, data and interpretations derived from the surveillance system are useful in setting public health priorities, planning and implementing control activities, and evaluating the effectiveness of interventions [4].

To manage the increasing sufferings from the effects of public health emergencies, the Ethiopian Public Health Institute established a fully integrated, adaptable and all-hazards approach system called Public Health Emergency Management (PHEM) system, which incorporated the International Health Regulation (IHR 2005) obligations. It is the process of emergency preparedness, early detection, response and recovering from the public health effects of emergency threats so that health, economic and environmental impacts are minimized [5, 6]. It primarily builds capacity at all surveillance levels, especially at the district level, with active community participation to detect early and respond to epidemics/other public health emergencies at a local level. Nationally, the surveillance system incorporated 23 priority diseases/conditions that are to be reported on immediately and weekly bases, based on the national guideline recommendation [5]. In addition to this, in the Amhara region, leishmaniasis and high HIV viral load (> 1000 copy/ml) are diseases/conditions under public health surveillance. Among those diseases under surveillance, polio and guinea worm are targeted for eradication; while neonatal tetanus, measles, leprosy, lymphatic filariasis, onchocerciasis and malaria are under elimination program. The community and health facilities especially health posts are the main sources of information for the surveillance. The information collected from this sites is compiled in standard forms, with simple analysis and then forwarded to the district health office. District level uses standard formats to compile, aggregate and send the data to zone/region using paper-based reporting formats, from which the central level receives. Feedback and information sharing follow the same route if any.

Thus, the implementation status of disease/event surveillance should be evaluated on regular bases to ensure that whether the system is serving a useful public health function or not and is meeting its objectives. This is important to improve the system’s usefulness, quality, efficiency, and attributes [4]. However, data on the performance of the existing surveillance system are scarce in the country, particularly in the study area. Therefore, we evaluated the performance of a public health surveillance system in Dangila district, northwest Ethiopia.

## Methods

### Study design and period

In August 2017, using 2016/17 as the base year, a concurrent embedded mixed quantitative/qualitative, facility-based cross-sectional study was conducted to assess the surveillance system performance status of Dangila district in line with its objectives.

### Study setting

The study was conducted in Dangila district, Awi zone, Amhara region, which is located at a distance of 482 km to the northwest of the capital city of Ethiopia, Addis Ababa. Based on the 2007 national census, the estimated population of the district in 2016/17 was 149,114. Of these, 77,212 (51.8%) were females [7]. The district has 29 kebeles served by 5 health centers and 29 health posts that provide primary health care service to the community including public health emergency management activities. In the study area, PHEM department is coordinated by two PHEM officers at the district level who leads the implementation of PHEM activities in all health centers and satellite health posts. Also, each health center has one PHEM focal person. Similarly, each satellite health post which is staffed by at least two health extension workers implement PHEM activities at the lower community level. Health care workers who are employed at health facilities are responsible for recording surveillance data on disease registries. Also, there is a standardized paper-based reporting format for immediately and weekly reportable diseases under surveillance. Thus, the surveillance system is fully integrated into the routine health care delivery system and designed to work 24 h a day and 365 days per year without any service interruption.

### Sample size and sampling technique

A total of 12 study units/sites were included in the study. Firstly, the district health office and all the five health centers were purposively included in the study to represent the district. Then, we included six (20%) health posts, at least one from each cluster health center, into the study with lottery methods. As a result, the qualitative part involved 12 key stakeholders interview (one from each site) that were selected purposively based on their experience, by thinking them as a rich source of information.

### Data collection

Data were collected using a semi-structured questionnaire adapted from updated CDC guideline for evaluating public health surveillance system through key stakeholder interviews and record reviews (Additional file 1). The questionnaire accessed issues concerning communication and reporting systems, availability of surveillance documentations, registers, reporting formats, data analysis and interpretation practices, computer skill and training profiles, epidemic response and preparedness situation, outbreak investigation and case confirmation, supervision and feedback system and questions on each surveillance attributes. Our data sources were surveillance reports, records, documents and key stakeholder interviews. The respondents were stakeholders (district PHEM officers, health center PHEM focal persons and health extension workers from health posts) from the study sites. When more than one stakeholders exist in the selected study sites we interviewed the most senior among them. The investigators were the data collectors.

### Data quality assurance

To minimize the subjectivity of responses from stakeholders, the investigators themselves participated as data collectors. The opinions and responses of stakeholders were cross-cheeked with facility records and reports to increase the accuracy of data. Observation was made on relevant documents like availability of guidelines, documentations, data analysis and feedback practices to compare and identify any data fallacy across different sources. During the session of each visit, we briefed the stakeholders about the purpose of the assessment, which was to evaluate the performance of the system and not merely the individual’s performances.

### Data analysis

All questionnaire responses were dichotomized as yes or no, except for the open-ended questions. Data were manually cleaned initially, and then the major qualitative findings were narrated and summarized based on thematic areas to supplement the quantitative findings. Microsoft Excel 2007 was used to analyze the quantitative findings.

## Operational definitions

### Acceptability

Willingness of surveillance stakeholders to implement the system as expressed by their active participation in case detection and reporting. It is measured quantitatively through reporting rates of health facilities for the past 12 months and timeliness of data reporting.

### Completeness

The proportion of health facilities that submitted a report to the higher level irrespective of the time of submission.

### Timeliness

The timeliness of the district was calculated by assessing how many of its expected reports have submitted within the prescribed time.

### Data quality

Was assessed based on content completeness of the reporting formats and validity of the data recorded.

### Positive predictive value

Is the proportion of cases detected by the surveillance case definition who actually have the disease being monitored.

### Flexibility

Is the ability of the system to adapt to changing needs such as the addition or removal of a new disease, the collection of additional data, modification of the reporting frequency, etc.

### Representativeness

Is the ability of the system to describe the occurrence and distribution of all reported cases accurately in terms of time, place and person

### Simplicity

Refers to the structure of the system and the ease of implementation while still meeting its objectives.

### Stability

Refers to the reliability (i.e., the ability to collect, manage, and provide data properly without failure) and availability (the ability to be operational when it is needed) of the public health surveillance system.

### Usefulness

Refers to the relevance of the system to surveillance stakeholders in terms of feeding information for action.

### Case detection

Is the process of identifying cases and outbreaks.

### Kebele

The lowest administrative unit next to a district with an estimated size of 1000 households.

### Surveillance attributes and indicators categorization

Once each investigator reviewed the data independently, the team has reached a common understanding on the implementation status of each surveillance attributes and indicators. Then, to summarize and facilitate an easy understanding of the results, the investigators decided and put their professional judgment (based on their professional expertise and national/international targets) on the implementation status of each surveillance attributes and indicators.

## Results

### Communication and reporting system

**Fig. 1:**
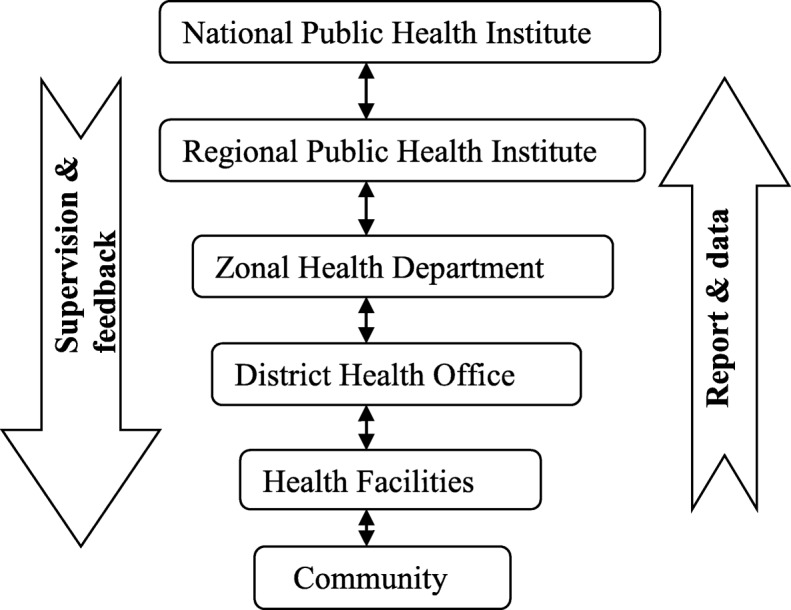
Diagram illustrating the formal and informal flow of surveillance data and feedbacks throughout the health system of Dangila district

Every Monday in the morning, the health posts prepare and send their weekly surveillance report to cluster health centers through a phone call. The health centers in turn aggregate and send the data they received from health posts to Dangila district health office on the same day afternoon. Similarly, the district health office receives reports from respective health centers with phone and sends it to Awi Zonal Health Department on Tuesday afternoon (Fig. 1). At the health post level, the sources of data for weekly reports were community representatives and disease registries. All health facilities and the district health office were using a standardized reporting format for data collection and aggregation purposes. Initially, report submission to the higher level was through a phone call, followed by the paper based report submission. Only one health center has a wired phone; the rest has been communicating with their mobile phone. In case of an emergency, the district health office communicates with the health facilities and Zonal Health Department on daily bases and the frequency of communication was on a weekly base in normal situations. None of the study sites were using email as a means of communication. As depicted in Fig. 1, surveillance data and information flows from the community to a higher level whereas supervision and feedback follow the reverse direction (Fig. 1).

### Availability of surveillance guidelines, documents, registers and formats

All health centers and the district health office had national guideline of PHEM, malaria, measles, polio, meningitis, neonatal tetanus, adverse effect following immunization surveillance, and reporting formats like weekly reporting form, line list, case investigation form, daily epidemic reporting form, case-based reporting form and they are using the guidelines properly. All health posts had “Health Workers Quick Guide for Public Health Emergency Management” guideline which is a comprehensive guideline primarily prepared for health extension workers in Amharic language. Besides, all study sites had a copy of each weekly surveillance reports in a file cabinet. On the other hand, surveillance officials were not using the appropriate rumor registration logbook.

### Case detection

All health facilities and the district health office had the case definitions for the diseases under eradication and elimination. Understanding of this case definitions at the visited health facilities was good as explained by PHEM focal persons and health extension workers working at health posts. However, the case definition for priority diseases was not posted on a wall or notice board. Health care workers were detecting any suspected cases of the reportable disease using standard case definitions. Malaria case detection was being conducted at a health center and health post level using Rapid Diagnostic Tests.

### Emergency preparedness and response

The district health office had Emergency Preparedness and Response Plan (EPRP), but not supported by the budget required to respond in case an emergency occurred. The rest health facilities didn’t have written EPRP as well as an outbreak investigation and supervision checklist. Neither the district health office nor the health centers had stocks of drugs and supplies for epidemic response. However, whenever an outbreak exists, health centers mobilize and/or purchase drugs and other medical supplies from their store which was reserved for routine services. All health centers and the district health office had a non-functional Rapid Response Team (RRT), but none of them had an emergency management committee and multi-sectorial PHEM task force. There was no car assigned to the public health emergency department only. No partner was working with the public health emergency department. Three out of the five health centers (60%) had trained PHEM focal person on basic PHEM, but none of the health extension workers took this training.

### Outbreak investigation and case confirmation

In 2016/17, no outbreak was detected and notified by the health facilities and the district health office. Consequently, shortage of emergency drugs and supplies was not encountered in the entire district in the year. However, health facilities didn’t have an outbreak investigation checklist for anticipated treats in the district.

### Data analysis and interpretation

Three out of the five (60%) health centers had at least one computer other than the health management information system (HMIS) and Smart Care computers, but none of them had used it for surveillance data entry and analysis purposes. But, there was no computer in the public health emergency management department of the district health office. All did data aggregation manually because of the gap in basic computer skills, computer inaccessibility, and less attention to it. All health facilities had denominators like total population disaggregated by sex and age, pregnancy status, malaria’s kebeles and so on, which are very important to describe the surveillance data in terms of time, place and persons. However, health centers simply send their weekly report to the district health office, which only includes the total number of cases, inpatient, deaths, and activities performed. In the same fashion, the district simply aggregates and sends the report to the higher level; no regular data analysis in terms of time, place and person. The threshold for action was set for malaria at the health facilities and district level.

### Supervision and feedback

All study sites had a supervision plan, but as mentioned by surveillance stakeholders it was not conducted regularly. Shortage of manpower, lack of resources and work overload were the common reasons given by stakeholders. All study sites didn’t have a well-prepared supervision checklist to assess PHEM activities in detail. But, there was an integrated checklist that contains few data elements of malaria and tuberculosis. Nonetheless, the district PHEM department has been supervised by higher bodies once in the year and one written feedback of supervision was given by the supervisory body. Despite this, we didn’t obtain a written letter of feedback sent to the lower level from the respective higher level. Generally, there was no regular feedback system, weekly bulletin preparation and dissemination regarding public health surveillance in the district and health facilities.

### Surveillance system attributes

#### Acceptability

In the district, the willingness and engagement of surveillance officials and reporting sites were as expected and the average reporting rates of health facilities were 96.3% as seen over the reporting weeks. Thus, the reporting system was acceptable by PHEM focal persons, PHEM officers and health extension workers. All the reporting health facilities were using the standard case definition for case detection and reporting was using the appropriate reporting formats.

#### Flexibility

The majority (83.3%) of stakeholders (PHEM focals, PHEM officer & HEWs) agreed that the current weekly reporting format can be used for new health events that are not listed in the nationally reportable diseases. This is because the reporting format has a blank column which says “Other” which means if any other events are there which needs to be included. And also, as explained by our stakeholders, it is possible to use technologies like the electronic reporting formats and to integrate with other systems.

#### Predictive value positive

To measure the sensitivity of the surveillance system, malaria data were taken as a representative of other reportable diseases under surveillance. Accordingly, all health centers and health posts reported that the current case definitions particularly malaria case definition is well stated in the way that can pick all malaria cases correctly. However, there were many false-positive cases reported in 2016/2017. During this period (July 2016–June 2017), a total of 17,208 suspected malaria fever cases were examined in Dangila district. Of which 1916 were confirmed cases with a positive predictive value of 11%.

#### Representativeness

As mentioned by stakeholders, the surveillance system can pick all public health emergencies in the whole community be it in the rural or urban areas irrespective of their age, sex, ethnicity, religion, and other social and economic status. Thus, the weekly public health emergency report comes from the lowest community organization level called 1 to 5 networks to the health posts; not only from health posts disease registries and then to the health centers. However, the weekly reporting format lacks some important variables like sex, age and other possible risk factors which are very important epidemiological variables that help to generate information to take appropriate actions.

#### Simplicity

Similarly, all stakeholders explained that the case definitions set for all country priority diseases are very clear and easy to understand. It has two types of case definitions; community and standard case definitions. The community case definition was very simple and interpreted by the local languages which could easily be used by those who can read Amharic language and is mainly be used by the health extension workers (HEWs). The reporting formats are also very easy to understand and fill data by all levels of health professionals and HEWs. It only needs 10–15 min to fill the form and is possible to update data on cases. It is also clear which disease to report, when to report, to whom to report and which reporting format to use. The laboratory confirmation for malaria takes 15–30 min, depending on whether microscopy or rapid diagnostic tests are used for testing. Moreover, conducting malaria and cholera rapid diagnostic tests at health facilities were found to be simple by health extension workers and PHEM focal persons.

#### The quality of data

All fields in the reporting formats were correctly filled and clear to read and understand. However, the variables date report received, WHO week, and the expected number of health facilities to report were the commonly missed variables. For malaria, as it is a weekly reportable disease, there was no data quality problem obtained. All health facilities and the district health office send their weekly report to the higher level as per the national guideline recommendation.

#### Stability

Of the 12 stakeholders interviewed, 8(66.7%) of them agreed that any restructuring didn’t affect the surveillance procedures and activities. The rest stakeholders accepted that staff turnover and lack of resource has affected the surveillance system to a lesser extent. Moreover, the surveillance system was found to be fully integrated into the existing health system and the operating resources for the system were fully covered by the local and regional governments.

#### Completeness

Completeness seems better as one goes up from health posts to cluster health centers and district health office, and it differs from one health facility to another health facility. But in reality, the incompleteness was masked as the data gets compiled at each steps. As mentioned above, even though the overall completeness of the district seemed very satisfying throughout the year (100%), as one goes down from the district health office to the health posts the reporting completeness becomes decreasing. Consequently, health centers’ annual average report completeness rate ranges from a minimum of 94.3% to a maximum of 98.2%.

#### Timeliness

**Fig. 2:**
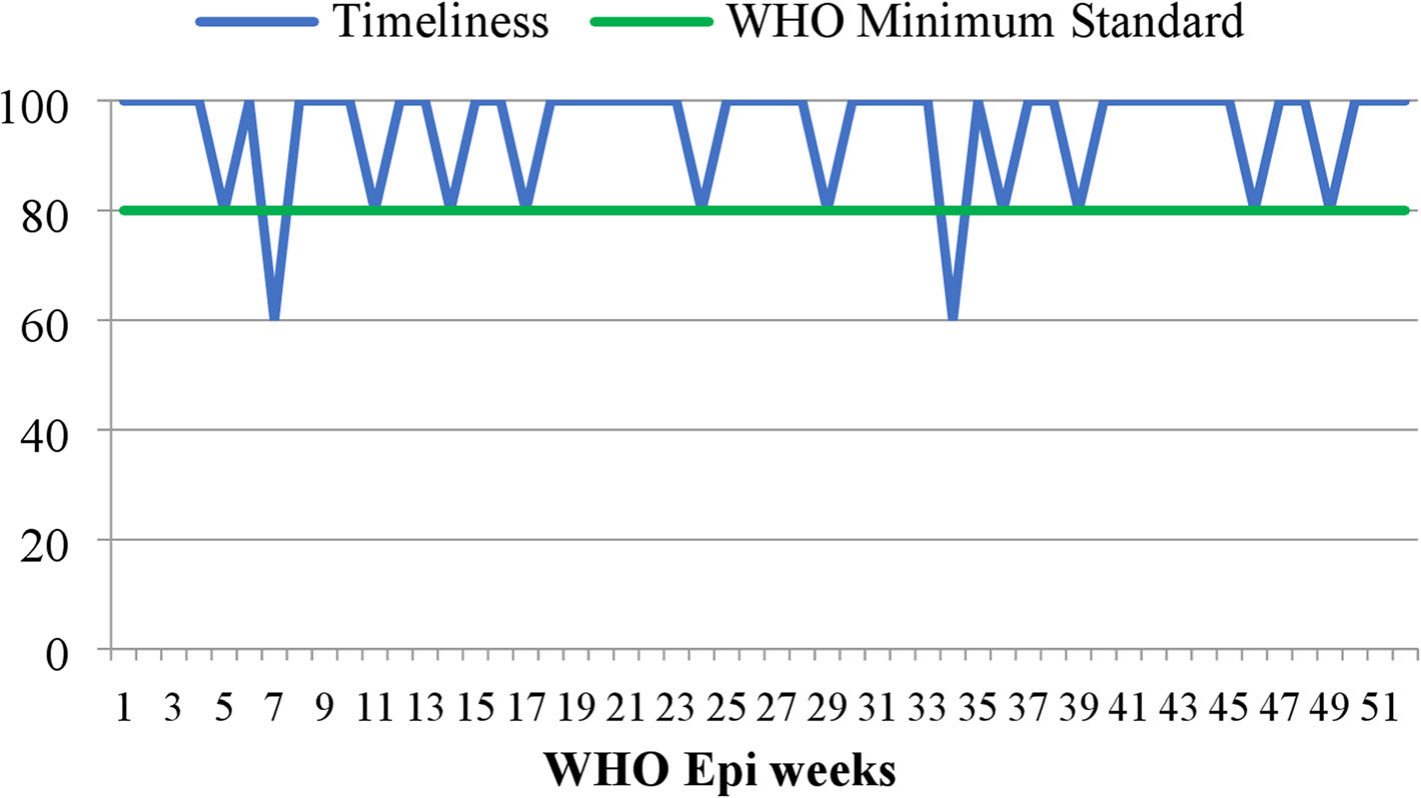
Timeliness of weekly surveillance report of Dangila district, northwest Ethiopia

As mentioned above, the reporting rates of health facilities were found to be satisfying irrespective of some reporting gaps at the health post level. But, of those that reported, the number of facilities that reported timely was difficult to calculate exactly, for the reason date report received was not recorded in all most all reports at the district health office level. But the district health office PHEM department has calculated the overall report timeliness of the district for each WHO week and the average timeliness was found to be 94.6% (Fig. 2).

#### Usefulness

The surveillance system was found to be very helpful to determine the magnitude of morbidity and mortality due to diseases under surveillance in the community as well as to assess the effectiveness of prevention and control measures for each priority diseases in the area. For the surveillance officials, it helped to detect outbreaks early and to diagnose it properly and thus to take action to prevent epidemics. The system also identified high priority areas for each disease under surveillance for resource allocation purposes. For example, the weekly malaria data enables surveillance officials to monitor malaria trends over time (using a malaria monitoring chart), to identify hot spot areas and to evaluate the effectiveness of malaria prevention and control activities.

Generally, as shown in Table 1 below, the authors tried to summarize the implementation status of each surveillance attributes. Accordingly, timeliness and completeness attributes were classified as very satisfactory, whereas surveillance data analysis and feedback system were found to be poorly implemented (Table 1).

**Table 1:** A summary of the implementation status of surveillance attributes and indicators in Dangila district, northwest Ethiopia

Surveillance performance indicators/attributes	Implementation status
Communication and reporting system	Satisfactory
Availability of surveillance guidelines, registers, documentations and formats	Satisfactory
Case detection	Satisfactory
Emergency preparedness and response	Unsatisfactory
Outbreak investigation and case confirmation	Unsatisfactory
Data analysis and interpretation	Very unsatisfactory
Supervision and feedback	Very unsatisfactory
Acceptability/participation	Satisfactory
Flexibility	Satisfactory
Sensitivity	Satisfactory
Representativeness	Satisfactory
Simplicity	Satisfactory
Data quality	Satisfactory
Stability	Satisfactory
Completeness	Very satisfactory
Timeliness	Very satisfactory
Usefulness	Satisfactory

## Discussion

According to the finding, the weekly surveillance report completeness in the district (100%), is higher than the 95% national target set by the country [8], as well as the 80% WHO target [9]; showing that all the visited health facilities were reporting to their respective level as per the standard in the national guideline. However, even though the overall completeness of the district seemed very satisfying throughout the year, it decreases as one goes down from the district health office to the health posts, because of the incompleteness was masked as the data gets compiled at each surveillance level.

Average report timeliness in the district was 94.6%, which is in line with the 95% national target set under the health sector transformation plan [8] and slightly lower than the 1st quarter timeliness report from Eritrea [10], but higher than the 61.5% timeliness report from upper east region of Ghana [11]. This timely report gives timely information for the district surveillance officials, which helps to detect and manage outbreaks early, to predict future outbreaks, trends of disease occurrence, cases for further studies, and action for problems identified on time.

Regarding surveillance communication, the structure of data flow from the lower to the upper level was well organized with a unidirectional flow of data, with simple and defined roles and responsibility of each reporting entity. However, the reporting flow has several obstacles such as inadequate infrastructure like the absence of transportation, lack of wired telephone, and no internet access for email service.

It is not enough to collect, record and report numerical data about morbidity, mortality and conditions from the catchment area; the data must also be analyzed in terms of time, place and person at each surveillance level where it is collected. However, the district health office simply merges and sends its weekly surveillance data to the Zonal Health Department, and subsequently to the Regional Public Health Institute; for the purpose of reporting to the respective higher level. This is because they are obligated to send the weekly report to the higher administrative level and not for real-time intervention of public health problems. The same is true for health centers; they aggregate the data they received from health posts and send it to the district health office every Monday in the afternoon. But, they didn’t analyze data on weekly, monthly and quarterly bases, except for malaria which was being monitored using the WHO malaria monitoring chart. As stated above, if the health facilities in the district don’t analyze and use the data, the utility of the surveillance system becomes minimal, which makes the system too weak to pick outbreaks early that could guide prompt response. The absence of data analysis, interpretation and utilization for local action seen in the present study is in line with the report from southwestern parts of the country [12], and the report from Nigeria and South Africa [13, 14]. The possible reasons might be a skill gap in data management system, weak supervision and feedback system, low or no legal enforcement to the surveillance activities, lack of incentives, lack of continues capacity building training, and lack of sense of ownership.

It is essential to build feedback loops into the system through regular epidemiological bulletins with tables and graphs showing trends and progress towards targets and reports on the investigation and control of outbreaks. However, in the assessed district, the feedback system was found to be weak due to the absence of epidemiologically analyzed and interpreted data to send it for health facilities; but verbal feedback was occasionally sent to the lower health facilities. Similar finding was reported from Akwaibom state of Nigeria [13]. Besides, even though the district was conducting a supportive supervisory activity in an irregular and integrated way, the working supervisory checklist was not well-prepared and it only talks about a few diseases under surveillance like malaria and tuberculosis. The reason for the absence of a holistic and detailed supervisory checklist could be lack of reference materials to prepare the checklist, less emphasis and commitment to prepare the checklist.

The district health office has Emergency Preparedness and Response Plan (EPRP) which was not supported by the budget and/ or logistics required to respond in case an emergency occurred. But, the district administrative council reserved an emergency budget which only be mobilized after an event has occurred. This slows down timely investigation and mitigation of expected events in the district by the district health office. The rest health facilities don’t have written EPRP as well as outbreak investigation and supervision checklist. Also, even though there is established rapid response team/ technical committee in the district and health facilities, it lacks functionality or regular monthly meeting at all levels. As observed in meeting minutes, the rapid response team had a meeting when an outbreak occurs, however, most of the team members were not trained on epidemic preparedness and response. Also, the rapid response team did not review their plans, actions, and learned experiences. This will make the district and the facilities to immediately wait and perceive the support of the higher levels in case of public health emergencies. This will make all responses to be late and give emergencies to take the chance and stay longer by adversely affecting the public. The misconception that a rapid response team is built to take immediate response once outbreaks happened might bet the possible reason for the non-functional rapid response team in the entire district.

Thus, during outbreaks, the team usually focuses on individual case treatment rather than investigating and directing response to the associated factors from the public point of view. In addition to these, the district annual performance review meeting didn’t address all the activities related to public health emergency surveillance and outbreak investigation and response. This shows as the emphasis given to disease/event surveillance was low in the study area.

Our mixed quantitative/qualitative study on public health surveillance system has some constraints. Like any performance assessments, Public Health Emergency (PHEM) personnel’s working within the public health surveillance system may be fearful of punitive measures or poor publicity if deficiencies were identified. However, during the sessions of each visit, we briefed stakeholders about the purpose of the assessment which was to evaluate the performance of the system and not merely the individual’s performances. Despite our attempts to brief the stakeholders, their responses may have been biased towards projecting a better image of their performance.

## Conclusions

The surveillance system is found to be simple, useful, flexible, acceptable, and representative. Weekly report timeliness and completeness are above the national and international targets. But, there remain problems with proper communication between surveillance levels, lack of budget, computer inaccessibility, lack of supervision checklist, masking of report completeness at lower levels and missed variables from weekly reporting format. Generally, the overall implementation status of the system in the district is not satisfactory to achieve the intended objective of surveillance for public health action due to the lack of regular data analysis, interpretation and feedback dissemination to which it may concerns. To strengthen and to create a well-functioning surveillance system, regular supportive supervision and epidemiologically analyzed and interpreted feedback system incorporating important variables is a must.

## Supplementary information

**Additional file 1:** Evaluation tools used for qualitative and quantitative data collection of the study.

## Data Availability

The datasets used and/or analyzed during the current study are available from the corresponding author up on reasonable request.

## Abbreviations

CDC: Communicable Disease Control
EPRP: Emergency Preparedness and Response Plan
HEWs: Health Extension Workers
PHEM: Public Health Emergency Management
WHO: World Health Organization
