# Supplementary material for: Evaluation of public health surveillance system performance in Dangila district, Northwest Ethiopia: a concurrent embedded mixed quantitative/qualitative facility-based cross-sectional study
Source: BMC Public Health. 2019 Oct 22;19:1343. doi: 10.1186/s12889-019-7724-y (PMC6805593; doi:10.1186/s12889-019-7724-y)
Supplement: Supplementary file 1 — Additional file 1: Evaluation tools used for qualitative and quantitative data collection of the study. [file 12889_2019_7724_MOESM1_ESM.docx]

**Questionnaire used to assess the district health office public health emergency management department**

**Background:**

Date-------------------Assessment team name: _____________________

Interviewer name: ___________________________________________

Respondent name & position: ___________________________________

Surveillance System: ______________________________

Catchment population _____________ kebele.......... HC.........HP........Hospitals......

Address: Office no _____________ Cell phone no _________ e-mail ________

**PART ONE:**

**A. Communication and reporting system assessment**

1. Which communication material did you have?  E-mail  wired phone  mobile  radio  fax  other--------------------------------

2. Did you have address of Health Center PHEM officers?  Yes  No

3. How frequently you communicate with the Health center PHEM officers on emergencies and other daily activities?  Daily  weekly  every 2 week monthly  quarterly  every 6 month  yearly  others----------------------

4. When are you expected to send weekly report to the zonal PHEM unit?  Monday  Tuesday  Wednesday  Thursday  Friday  Saturday  Sunday

5. When are you expected to receive weekly report from HCs?  Monday  Tuesday  Wednesday  Thursday  Friday  Saturday  Sunday

6. How is the zonal PHEM communicating the woreda PHEM officers in case of immediately reportable diseases?  by e-mail  by phone  by fax  regular weekly report  others

7. Did you send summary or short report to the administrative /program leaders or other responsible organs on planning, prevention and control activities addressing Important issues at community level that have arisen through the surveillance system?  Yes  No

8. If answer for Q7 is yes to whom did you send? ----------------------------------------------------

**B. Assessment of availability of Surveillance Documentation, Registers, and Forms**

1. Is there a national manual for malaria and cholera surveillance?  Yes  No □NA

2. Did you have National Guide line for PHEM?  Yes  No  Not Applicable

3. Did you have standard case definition for all country priority diseases? (Malaria, Cholera)  Yes  No  NA

4. Was the case definition posted?  Yes  No

5. If answer for Q4 is No, for which disease(s) did you lack the case definition?

6. Did you have case based reporting formats for out breaks?  Yes  No  NA

7. Was there guide line for specimen collection, handling and transportation to the next level?  Yes  No  NA

8. Did you have line list for reporting outbreaks?( malaria, cholera)  Yes  No  Not Applicable

**C. Data analysis, Computer skill and training assessment**

1. Had you trained on surveillance system?  Yes  No

2. If answer for Q1 is yes a) when----------- ? b) Topic----------------------------? c) For how long? -------------

3. Did you give any onsite orientation about surveillance system for HC and HP PHEM focal persons?  Yes  No

4. Was data compiled?  Yes  No

5. Did you have computer?  Yes  No

6. Is it functional)?  Yes  No

7. How the data entry and compilation is accomplished?  Manual  Computer  other-

8. Did you have computer skill on  Msword  Msexcel  MS power point  Epi-info?

9. Did you analyze data of the surveillance system?  Yes  No

10. If answer for Q9 is yes, did you describe data by time, place, and person: □Yes □No

11. Did you have denominators for data analysis?  total pop  male  female  <5yrs

12. Please indicate the frequency of your data analysis.  weekly  every two week Monthly  quarterly every 6 month  annually  No regular time

13. Did you notify the results of your analysis to the higher level PHEM?  Yes  No

14. Did you notify the results of your analysis to the lower level PHEM?  Yes  No

**D. Epidemic response and preparedness assessment**

1. Did you have plan for epidemic response and preparedness?  Yes  No

2. Did you have emergency stocks of drugs and supplies?  Yes  No

3. If answer for Q2 is No, how did you control epidemics? -----------------------------------------

4. Had you experienced shortage of drugs, vaccines and supplies in 2009 EFY?  Yes  No

5. Was an epidemic management committee built in your office?  Yes  No  Not Applicable

6. Did the epidemic management committee have regularly scheduled meeting time?  Yes  No

7. Was Rapid response team (RRT) built in your office?  Yes  No  Not Applicable

8. Did the RRT have regularly scheduled meeting time during epidemics?  Yes  No

9. Did you have case management protocol for epidemic prone diseases?  Yes No  Not Applicable

10. Did your PHEM have multi sectoral emergency preparedness and response task force?  Yes  No  Not Applicable

11. Were partners working together with your office on emergencies?  Yes  No

12. If answer for Q11 is yes, what type of supports did they give to your office?

--------------------------------------------------------------------------------------------------

13. Was there a budget for epidemic response?  Yes  No

14. Who had the authority to mobilize the emergency finance? Health office head  PHEM case team leader  PHEM officer  other-----------------------------------------------------------------------

15. Had you a vehicle assigned for emergencies (PHEM)?  Yes  No  Not applicable

16. If answer for Q15 is No, how did you address emergencies?

**E. Outbreak investigation and case confirmation assessment**

1. Had you investigated any outbreak in 2009 EFY?  Yes  No, list if any

2. Did you have outbreak investigation check list?  Yes  No

3. If answer for Q2 is No, how did you know possible factors for the outbreak?

---------------------------------------------------------------------------------------------------------------

4. Where was laboratory confirmation of cases?  regional lab  Hospital  EHNRI  HC  other----------------------

5. Who was responsible to investigate an outbreak?  RRT  HEWs  Health office staffs experts organized randomly  health facility staffs  other--------------------------

6. Had you faced any challenge in outbreak investigation in 2009 EFY?  Yes  No

7. If answer for Q6 is yes,

a) List the challenges -----------------------------------------------------------------------------------------------

b) List the alternatives that you take to tackle the challenges -----------------------------------------------

**F. Supervision and feedback assessment**

1. Did you have supervision plan in 2009 EFY?  Yes  No

2. If answer for Q1 is No, how did you supervise? -----------------------------------------------------------------------

3. If for Q1 is yes, did you supervise the HCs and HPs according to your plan in 2009 EFY?  Yes  No

4. If answer for Q3 is No, what is the reason? ------------------------------------------------------------------

5. If answer for Q3 is yes, how many times did you supervise each HC and HP in 2009 EFY? ______

6. Had you reviewed about surveillance practice by higher level supervision?  Yes  No

7. Did you have regular supervision checklist?  Yes  No

8. If answer for Q7 is No, how did you supervise the health facilities & Health posts? -------------------

9. Were you supervised by higher level officers in 2009 EFY?  Yes  No

10. If answer for Q9 is yes how many times in 2009 EFY? -------------------------

11. Did you send feedback of your supervision to the health centers (HCS) and health posts (HPs) commenting/indicating their strong and weak sides?  Yes  No

12. If answer for Q11 is No, why? --------------------------------------------------------------------------------

13. If answer for Q11 is yes, for how many HCs and HPs did you send a feedback in 2009 EFY____________________________

14. Had you received feedback from higher level supervisors in 2009 EFY?  Yes  No

15. If answer for Q14 is yes how many feedbacks did you received in 2009 EFY? --------------

16. Had you faced any challenge on supervision and feedback in 2009 EFY?  Yes  No

**PART-TWO**

**Is The Surveillance System Helpful?**

1. To detect outbreaks early on time to permit accurate diagnosis?  Yes  No

2. To estimate the magnitude of morbidity and mortality?  Yes  No

3. Permit assessment of the effect of prevention and control programs?  Yes  No

4. To estimate research intended to lead to prevention and control?  Yes  No

**Describe Each System Attributes:**

**I. Simplicity:**

1. Is the case definition easy for case detection by all level health professionals?  Yes  No

2. Does the surveillance system allow all levels of professionals to fill data?  Yes  No

3. Does the surveillance system help to record and report data on time?  Yes  No

4. Does the surveillance system have necessary information for investigation?  Yes  No

5. Does the surveillance system allow updating data on the cases?  Yes  No

6. How long does it take to fill the format?  <5 min 5 to 10 min  10 to15min  >15 min

7. How long does it take to have laboratory confirmation? ------------------------------------------

**II. Flexibility**

1. Can the current reporting formats be used for other newly occurring health event (disease) without much difficulty?  Yes  No

2. Did you think that any change in the existing procedure of case detection and reporting formats will be difficult to implement?  Yes  No, Add your explanation -----------------------------------

3. Is the system easy to add new variables?  Yes  No

4. Is the surveillance system easy to integrate with other systems?  Yes  No

5. Is the surveillance system easy to add new disease on report?  Yes  No

6. Is the system easy to add new information technology?  Yes  No

**III. Data quality**

1. Are all reported forms Complete?  Yes  No

2. If answer for Q1 is No, how many unfilled spaces are in your 2009 EFY report? -------------3. Percentage of unknown or blank responses to variables from the total reports of 2009 EFY report--_____________________________________________

4. Percent of reports which are complete (that is with no blank or unknown responses) from the total reports ---------------

5. Is the recorded data clear to read and understand?  Yes  No

6. If answer for Q5 is No, how many records are not clear/are difficult to understand in 2009 EFY report? -------------------------------------------

7. Percent of records which are difficult to read/ understand. --------------------------

**IV. Acceptability**

1. Do you think all the reporting agents accept and well engaged to the surveillance activities?  Yes  No

**2.** If yes, how many are active participants (of the expected)? -----------

**3.** If No, what is the reason for their poor participation in the surveillance activity?

A) Lack of understanding of the relevance of the data to be collected

B) No feedback / or recognition given by the higher bodies for their contribution

C) Reporting formats are difficult to understand

D) Report formats are time consuming

E) Other: -----------------------------------------------------

**4.** Were all participants using the standard case definition to identify cases?  Yes  No

**5.** Were all the reporting agents send their report using the current and appropriate surveillance reporting format?  Yes  No

**6.** Were all the health professionals aware about the surveillance system?  Yes  No

**7.** Was all PHEM officers send report on time?  Yes  No

**V. Representativeness**

1. Was the surveillance system enabled to follow the health and health related events in the whole community?  Yes  No

2. If answer for Q1 is no, who do you think is well benefited by the surveillance system?  The urban  the rural  both

3. Are all the Socio demographic variables included in the surveillance reporting format?  Yes  No

4. If the answer for Q3 is No, which a) Sex---- b) age group---C) ethnic group----d) religion---- is less represented?

**VI. Timeliness**

1. Are all reporting sites reporting on time?  Yes  No

2. Percent of reporting sites that report on time. --------------

**VII. Completeness**

*1.* Are all reporting sites reporting?  Yes  No

*2.* Percent of Health centers& Health posts that send report of each week in 2009 EFY. -------

**VIII. Stability**

1. Was any new restructuring affected the procedures and activities of the surveillance?  Yes  No

2. Was there lack of resources that interrupt the surveillance system?  Yes  No

3. Was there any time /condition in which the surveillance is not fully operating?  Yes  No

4. If the answer for Q3 is yes, explain why? ---------------------------------------------------------------------------------------------------------------------------------------------------------------------------------------------

**Questionnaire used to assess health centers**

**BACKGROUND:**

Town/woreda _______________ HC Name________________ No. of HPs-----------------

Catchment population ________________

Respondent(s) ____________________________________________________

Address: Office no _________ Cell phone no __________e-mail ____________

**PART ONE:**

**A. Communication and reporting system assessment**

1. Which communication material did you have?  E-mail  wired phone  mobile  radio  fax  other--------------------------------

2. Did you have address of Health Bureau/zonal/woreda PHEM officers?  Yes  No

3. How frequently you communicate with the Health Bureau/zonal PHEM officers on emergencies and other daily activities?  Daily  weekly  every 2 week monthly  quarterly  every 6 month  yearly  others-------------------------

4. Did you have address of HP HEWs?  Yes  No

5. How frequently you communicate with the HP HEWs on emergencies and other daily activities?  daily  weekly  every 2 week monthly  quarterly  every 6 month  yearly  others------------------------------------------------

6. When are you expected to send weekly report to the woreda PHEM Unit?  Monday  Tuesday  Wednesday  Thursday  Friday  Saturday  Sunday

7. When are you expected to receive weekly report from HPs?  Monday  Tuesday  Wednesday  Thursday  Friday  Saturday  Sunday

8. How is the Health Center communicating the HPs HEWs in case of immediately reportable diseases?  by e-mail  by phone  by fax  regular weekly report  others

9. Did you send summary or short report to the administrative /program leaders or other responsible organs on planning, prevention and control activities addressing important issues at community level that have arisen through the surveillance system?  Yes  No

10. If answer for Q9 is yes to whom did you send? ---------------------------------------------------------------

**B .Assessment of availability of Surveillance Documentation, Registers, and Forms**

1. Did you have National Guide line for PHEM?  Yes  No  Not Applicable

2. Did you have standard case definition for all country priority diseases?  Yes  No  NA

3. Was the case definition posted?  Yes  No

4. If answer for Q3 is No, for which disease(s) did you lack the case definition?

5. Did you have case based reporting formats for out breaks?  Yes  No  NA

6. Was there national manual for surveillance?  Yes  No  NA

7. Was there guide line for specimen collection, handling and transportation to the next level?  Yes  No  NA

8. Did you have line list for reporting outbreaks?  Yes  No  Not Applicable

**C .Data analysis, Computer skill and training assessment**

1. Had you trained on surveillance system?  Yes  No

2. If answer for Q1 is yes a) when-----------? b) Topic----------------------------? c) For how long? ------------

3. Did you give any onsite orientation about surveillance system for HC and HP PHEM focal persons?  Yes  No

4. Was data compiled?  Yes  No

5. Did you have computer?  Yes  No

6. Is it functional)?  Yes  No

7. How the data entry and compilation is accomplished?  Manual  Computer  other-

8. Did you have computer skill on  Ms word  Ms excel  MS power point  Epi-info

9. Did you analyze data of the surveillance system?  Yes  No

10. If answer for Q9 is yes, did you describe data by time, place and person: □Yes □No

11. Did you have denominators for data analysis? total pop male female  <5

12. Please indicate the frequency of your data analysis.  weekly  every two week Monthly  quarterly every 6 month  annually  No regular time

13. Did you notify the results of your analysis to the higher level PHEM?  Yes  No

14. Did you notify the results of your analysis to the lower level PHEM?  Yes  No

**D .Epidemic response and preparedness assessment**

1. Did you have plan for epidemic response and preparedness?  Yes  No

2. Did you have emergency stocks of drugs and supplies?  Yes  No

3. If answer for Q2 is No, how did you control epidemics? ------------------------------------------

4. Had you experienced shortage of drugs, vaccines and supplies in 2009 EFY?  Yes  No

5. Was an epidemic management committee built in your office?  Yes  No  Not Applicable

6. Did the epidemic management committee have regularly scheduled meeting time?  Yes  No

7. Was Rapid response team (RRT) built in your office?  Yes  No  Not Applicable

8. Did the RRT have regularly scheduled meeting time during epidemics?  Yes  No

9. Did you have case management protocol for epidemic prone diseases?  Yes No  Not Applicable

10. Did your PHEM have multi sectoral emergency preparedness and response task force?  Yes  No  Not Applicable

11. Were partners working together with your office on emergencies?  Yes  No

12. If answer for Q11 is yes, what type of supports did they give to your office?

13. Was there a budget for epidemic response?  Yes  No

14. Who had the authority to mobilize the emergency finance? Health center head  experts  other-------------------------------------------------------------------------

15. Had you a car assigned for emergencies (PHEM)?  Yes  No  Not applicable

16. If answer for Q15 is NO, how did you address emergencies?

**E .Outbreak investigation and case confirmation assessment**

1. Had you investigated any outbreak in 2009EFY?  Yes  No, list if any

2. Did you have outbreak investigation check list?  Yes  No

3. If answer for Q2 is No, how did you know possible factors for the outbreak?

4. Where was laboratory confirmation of cases? regional lab Hospital EHNRI  HC  other----------------------

5. Who was responsible to investigate an outbreak?  RRT  HEWs  staffs of Health Bureau experts organized randomly  health facility staffs  other-------------------------------

6. Had you faced any challenge in outbreak investigation in 2009 EFY?  Yes  No

7. If answer for Q7 is yes,

a) List the challenges -----------------------------------------------------------------------------------------------b) List the alternatives that you take to tackle the challenges -----------------------------------------------

**F. Supervision and feedback assessment**

1. Did you have supervision plan in 2009 EFY?  Yes  No

2. If answer for Q1 is No, how did you supervise? -------------------------------------------------------------

3. If for Q1 is yes, did you supervise the HPs according to your plan in 2009 EFY?  Yes  No

4. If answer for Q3 is No, what is the reason? ---------------------------------------------------------

5. If answer for Q3 is yes, how many times did you supervise each HP in 2009 EFY? ______

6. Had you reviewed about surveillance practice by higher level supervision?  Yes  No

7. Did you have regular supervision checklist?  Yes  No

8. If answer for Q7 is No, how did you supervise the HPs? ---------------------------

9. Were you supervised by higher level officers in 2009 EFY?  Yes  No

10. If answer for Q9 is yes how many times in 2009 EFY? -------------------------

11. Did you send feedback of your supervision to the health posts commenting/indicating their strong and weak sides?  Yes  No

12. If answer for Q11 is No, why? ------------------------------------------------------------------------------

13. If answer for Q11 is yes, for how many HPs did you send a feedback in 2009 EFY_____

14. Had you received feedback from higher level supervisors in 2009 EFY?  Yes  No

15. If answer for Q14 is yes how many feedbacks did you received in 2009 EFY? ------------------

16. Had you faced any challenge on supervision and feedback in 2009 EFY?  Yes  No

**PART-TWO**

**Is The Surveillance System Helpful?**

1. To detect outbreaks early on time to permit accurate diagnosis?  Yes  No

2. To estimate the magnitude of morbidity and mortality?  Yes  No

3. Permit assessment of the effect of prevention and control programs?  Yes  No

4. To estimate research intended to lead to prevention and control?  Yes  No

**Describe Each System Attributes:**

**1. Simplicity:**

1. Is the case definition easy for case detection by all level health professionals?  Yes  No

2. Does the surveillance system allow all levels of professionals to fill data?  Yes  No

3. Does the surveillance system help to record and report data on time?  Yes  No

4. Does the surveillance system have necessary information for investigation?  Yes  No

5. Does the surveillance system allow updating data on the cases?  Yes  No

6. How long does it take to fill the format?  <5 min 5 to 10 min  10 to15min  >15 min

7. How long does it take to have laboratory confirmation? ---------------------------------------------

**2. Flexibility**

1. Can the current reporting formats be used for other newly occurring health event (disease) without much difficulty?  Yes  No

2. Did you think that any change in the existing procedure of case detection and reporting formats will be difficult to implement?  Yes  No, Add your explanation -----------------------------------

3. Is the system easy to add new variables?  Yes  No

4. Is the surveillance system easy to integrate with other systems?  Yes  No

5. Is the surveillance system easy to add new disease on report?  Yes  No

6. Is the system easy to add new information technology?  Yes  No

**3. Data quality**

1. Are all reported forms Complete?  Yes  No

2. If answer for Q1 is No, how many unfilled spaces are in your 2009 EFY report? ---------------

3. Percentage of unknown or blank responses to variables from the total reports of 2009 EFY report--___________________

4. Percent of reports which are complete that is with (no blank or unknown responses) from the total reports ---------------

5. Is the recorded data clear to read and understand?  Yes  No

6. If answer for Q5 is No, how many records are not clear/are difficult to understand in 2009 EFY report? -

7. Percent of records which are difficult to read/ understand. ---------------------------------------

**4. Acceptability**

1. Do you think all the reporting agents accept and well engaged to the surveillance activities?  Yes  No

2. If yes, how many are active participants (of the expected)? -----------

3. If No, what is the reason for their poor participation in the surveillance activity?

A) Lack of understanding of the relevance of the data to be collected

B) No feedback / or recognition given by the higher bodies for their contribution

C) Reporting formats are difficult to understand

D) Report formats are time consuming

E) Other: -----------------------------------------------------

4. Were all participants using the standard case definition to identify cases?  Yes  No

5. Were all the reporting agents send their report using the current and appropriate surveillance reporting format?  Yes  No

6. Were all the health professionals aware about the surveillance system?  Yes  No

7. Was all PHEM officers send report on time?  Yes  No

**5. Representativeness**

1. Was the surveillance system enabled to follow the health and health related events in the whole community?  Yes  No

2. If answer for Q1 is no, who do you think is well benefited by the surveillance system?  The urban  the rural  both

3. Are all the Socio demographic variables included in the surveillance reporting format?  Yes  No

4. If the answer for Q3 is No, which a) Sex---- b) age group---C) ethnic group----d) religion---- is less represented?

**6. Timeliness**

1. Are all reporting sites reporting on time?  Yes  No

2. Percent of reporting sites that report on time. --------------

**7. Completeness**

1. Are all reporting sites reporting?  Yes  No

2. Percent of HPs that send report of each week in 2009 EFY. ------------------

**8. Stability**

1. Was any new restructuring affected the procedures and activities of the surveillance?  Yes  No

2. Was there lack of resources that interrupt the surveillance system?  Yes  No

3. Was there any time /condition in which the surveillance is not fully operating?  Yes  No

4. If the answer for Q3 is yes, explain why? ------------------------------------------------------------------------------------------------------------------------------------------------------------------------------------

**Questionnaire used to assess health posts**

**BACKGROUND:**

Town/kebele________________No. of HAD …………… No. of 1 to 5 Networks…………

Health Post________________________

Catchment population ______________________

Respondent(s)_______________________________________________________

Address: Office no _______________ Cell phone no _____________ e-mail

**PART ONE:**

**A .Communication and reporting system assessment**

1. Which communication material did you have?  E-mail  wired phone  mobile  radio  fax  other--------------------------------

2. Did you have address of Health center PHEM officers?  Yes  No

3. How frequently you communicate with the Health center PHEM officers on emergencies and other daily activities?  Daily  weekly  every 2 week monthly  quarterly  every 6 month  yearly  others-------------------

4. When are you expected to send weekly report to the Health center PHEM unit?  Monday  Tuesday  Wednesday  Thursday  Friday  Saturday  Sunday

5. How is the Health post communicating the HCs PHEM officers in case of immediately reportable diseases?  by e-mail  by phone  by fax  regular weekly report  others

6. Did you send summary or short report to the administrative /program leaders or other responsible organs on planning, prevention and control activities addressing Important issues at community level that have arisen through the surveillance system?  Yes  No

7. If answer for Q6 is yes to whom did you send? ---------------------------------------------------

**B .Assessment of availability of Surveillance Documentation, Registers, and Forms**

1. Did you have National Guide line for PHEM?  Yes  No  Not Applicable

2. Did you have standard case definition for all country priority diseases?  Yes  No  NA

3. Was the case definition posted?  Yes  No

4. If answer for Q2 is No, for which disease(s) did you lack the case definition?

5. Did you have case based reporting formats for out breaks?  Yes  No  NA

6. Was there national manual for surveillance?  Yes  No  NA

7. Was there guide line for specimen collection, handling and transportation to the next level?  Yes  No  NA

8. Did you have line list for reporting outbreaks?  Yes  No  Not Applicable

**C. Training assessment**

1. Had you trained on surveillance system?  Yes  No

2. If answer for Q1 is yes a) when-----------? b) Topic----------------------------? c) For how long?

**D. Epidemic response and preparedness assessment**

1. Did you have plan for epidemic response and preparedness?  Yes  No

2. Did you have emergency stocks of drugs and supplies?  Yes  No

3. If answer for Q2 is No, how did you control epidemics? ---------------------------------------------------

4. Had you experienced shortage of drugs, vaccines and supplies in 2009 EFY?  Yes  No

5. Was an epidemic management committee built in your office?  Yes  No  Not Applicable

6. Did the epidemic management committee have regularly scheduled meeting time?  Yes  No

7. Was Rapid response team (RRT) built in your office?  Yes  No  Not Applicable

8. Did the RRT have regularly scheduled meeting time during epidemics?  Yes  No

9. Did you have case management protocol for epidemic prone diseases?  Yes No  Not Applicable

10. Was there a budget for epidemic response?  Yes  No

11. Who had the authority to mobilize the emergency finance? Health post staffs  Health center staffs  other-------------------------------------------------------------------------

12. Had you a car assigned for emergencies (PHEM)?  Yes  No  Not applicable

13. If answer for Q12 is No, how did you address emergencies?

**E .Outbreak investigation and case confirmation assessment**

1. Had you investigated any outbreak in 2009 EFY?  Yes  No, list if any

2. Did you have outbreak investigation check list?  Yes  No

3. If answer for Q2 is No, how did you know possible factors for the outbreak?

------------------------------------------------------------------------------------------------------------------------

4. Where was laboratory confirmation of cases?  regional lab  Hospital  EHNRI  HC  other----------------------

5. Who was responsible to investigate an outbreak?  RRT  HEWs  staffs of health bureau  experts organized randomly  health center staffs  other-------------------------------------

6. Had you faced any challenge in outbreak investigation in 2009 EFY?  Yes  No

7. If answer for Q6 is yes,

a) List the challenges ----------------------------------------------------------------------------------------------

**Field Supervision and feedback assessment**

1. Were you supervised by higher level officers in 2009 EFY?  Yes  No

2. If answer for Q1 is yes how many times in 2009 EFY? -------------------------

3. Had you received feedback from higher level supervisors in 2009 EFY?  Yes  No

4. If answer for Q3 is yes how many feedbacks did you received in 2009 EFY? -----------------

5. Had you faced any challenge on supervision and feedback in 2009 EFY?  Yes  No

a) what?___________________________________________________________

**PART-TWO**

**Is The Surveillance System Helpful?**

1. To detect outbreaks early on time to permit accurate diagnosis?  Yes  No

2. To estimate the magnitude of morbidity and mortality?  Yes  No

3. Permit assessment of the effect of prevention and control programs?  Yes  No

4. To estimate research intended to lead to prevention and control?  Yes  No

**Describe Each System Attributes:**

**1. Simplicity:**

1. Is the case definition easy for case detection by all level health professionals?  Yes  No

2. Does the surveillance system allow all levels of professionals to fill data?  Yes  No

3. Does the surveillance system help to record and report data on time?  Yes  No

b) List the alternatives that you take to tackle the challenges ------------------------------------------

4. Does the surveillance system have necessary information for investigation?  Yes  No

5. Does the surveillance system allow updating data on the cases?  Yes  No

6. How long does it take to fill the format?  <5 min 5 to 10 min  10 to15min  >15 min

7. How long does it take to have laboratory confirmation? -----------------------------------------

**2. Flexibility**

1. Can the current reporting formats be used for other newly occurring health event (disease) without much difficulty?  Yes  No

2. Did you think that any change in the existing procedure of case detection and reporting formats will be difficult to implement?  Yes  No, Add your explanation -------------------------------------------

3. Is the system easy to add new variables?  Yes  No

4. Is the surveillance system easy to integrate with other systems?  Yes  No

5. Is the surveillance system easy to add new disease on report?  Yes  No

6. Is the system easy to add new information technology?  Yes  No

**3. Data quality**

1. Are all reported forms Complete?  Yes  No

2. If answer for Q1 is No, how many unfilled spaces are in your 2009EFY report? -------------

3. Percentage of unknown or blank responses to variables from the total reports of 2009 EFY report--__________________________________

4. Percent of reports which are complete (that is with no blank or unknown responses) from the total reports ---------------

5. Is the recorded data clear to read and understand?  Yes  No

6. If answer for Q5 is No, how many records are not clear/are difficult to understand in 2009 EFY report? --------

7. Percent of records which are difficult to read/ understand. ------------------------------------------

**4. Acceptability**

**1.** Do you think all the reporting agents accept and well engaged to the surveillance activities?  Yes  No

**2.** If yes Q1, how many are active participants (of the expected)? -----------

**3.** If No Q1, what is the reason for their poor participation in the surveillance activity?

A) Lack of understanding of the relevance of the data to be collected

B) No feedback / or recognition given by the higher bodies for their contribution

C) Reporting formats are difficult to understand

D) Report formats are time consuming

E) Other: -----------------------------------------------------

**4.** Were all participants using the standard case definition to identify cases?  Yes  No

**5.** Were all the reporting agents send their report using the current and appropriate surveillance reporting format?  Yes  No

**6.** Were all the HEWs& health professionals aware about the surveillance system?  Yes  No

**7.** Was HEWs send report on time?  Yes  No

**5. Representativeness**

1. was the surveillance system enabled to follow the health and health related events in the whole community?  Yes  No

2. If answer for Q1 is no, who do you think is well benefited by the surveillance system?  The urban  the rural  both

3. Are all the Socio demographic variables included in the surveillance reporting format?  Yes  No

4. If the answer for Q3 is No, which a) Sex---- b) age group---C) ethnic group----d) religion---- is less represented?

**6. Timeliness**

1. Are HEWs reporting on time?  Yes 

**7. Completeness**

1. Are all reporting sites reporting?  Yes  No

2. Percent of HPs that send report of each week in 2009 EFY. ------------------

**8. Stability**

1. Was any new restructuring affected the procedures and activities of the surveillance?  Yes  No

2. Was there lack of resources that interrupt the surveillance system?  Yes  No

3. Was there any time /condition in which the surveillance is not fully operating?  Yes  No

4. If the answer for Q3 is yes, explain why? ------------------------------------------------------------------------------------------------------------------------------------------------------------------------------------
